# Gray Matter Pathology in MS: Neuroimaging and Clinical Correlations

**DOI:** 10.1155/2013/627870

**Published:** 2013-06-25

**Authors:** Justin Morris Honce

**Affiliations:** Radiology Department, Division of Neuroradiology, University of Colorado Denver, 12700 E 19th Avenue Mail Stop C278, Aurora, CO 80045, USA

## Abstract

It is abundantly clear that there is extensive gray matter pathology occurring in multiple sclerosis. While attention to gray matter pathology was initially limited to studies of autopsy specimens and biopsies, the development of new MRI techniques has allowed assessment of gray matter pathology *in vivo*. Current MRI techniques allow the direct visualization of gray matter demyelinating lesions, the quantification of diffuse damage to normal appearing gray matter, and the direct measurement of gray matter atrophy. Gray matter demyelination (both focal and diffuse) and gray matter atrophy are found in the very earliest stages of multiple sclerosis and are progressive over time. Accumulation of gray matter damage has substantial impact on the lives of multiple sclerosis patients; a growing body of the literature demonstrates correlations between gray matter pathology and various measures of both clinical disability and cognitive impairment. The effect of disease modifying therapies on the rate accumulation of gray matter pathology in MS has been investigated. This review focuses on the neuroimaging of gray matter pathology in MS, the effect of the accumulation of gray matter pathology on clinical and cognitive disability, and the effect of disease-modifying agents on various measures of gray matter damage.

## 1. Background

Multiple sclerosis (MS) is a chronic inflammatory demyelinating disease of the central nervous system (CNS); focal areas of white matter demyelination have long been considered the key feature of MS [1]. Despite this belief that MS is primarily a white matter disease, early pathologic studies had reported focal areas of cortical demyelination in MS patients [2, 3]. In 1962, Brownell and Hughes [4] showed that, in MS, cortical demyelinating lesions represented up to 26% of the total number of cerebral plaques. Despite these early indications of the cortical pathology occurring in MS, in general very little attention was paid to cortical pathology. It is likely that this was due to both the difficulty in identifying cortical lesions at autopsy via conventional histochemical techniques and the marked conspicuity of inflammatory lesions in the white matter [5]. This focus on white matter demyelination rather than cortical pathology was initially compounded with the advent of MRI: conventional MRI techniques for imaging MS identify a majority of focal white matter lesions but are very insensitive for the detection of cortical MS lesions [6]. 

Despite initial focus on white matter demyelination, there has been increasing focus on the gray matter pathology occurring in MS. This shift in focus was spurred by new immunohistochemical techniques which markedly increased the visibility of cortical lesions *ex vivo* [7, 8]. Improved *ex vivo* detection of gray matter lesions spurred work to improve the *in vivo* detection of gray matter pathology with MRI. As a result, new MRI imaging modalities are being utilized to provide greater insight into both the spatiotemporal pattern and distribution of gray matter pathology in MS. *In vivo* evaluation of the impact of gray matter pathology on disability and cognition and the effect of disease-modifying therapies on the accumulation of gray matter pathology are now ongoing. While many questions have yet to be fully answered, the importance of gray matter pathology in MS is clear, and it has taken the center stage in MS-related research.

This review focuses on imaging gray matter pathology in MS and addresses the relationship between measures of gray matter pathology and the clinical and cognitive status of MS patients. First, the pathologic classification and theories of pathogenesis of gray matter pathology are reviewed. We then detail the current imaging techniques utilized for both the detection of cortical demyelination and quantitation of both diffuse gray matter damage and gray matter atrophy. Finally, we discuss the clinical impact of gray matter pathology on disability and the effect of disease-modifying therapies on measures of gray matter pathology.

## 2. Pathologic Description of Gray Matter Pathology in MS

### 2.1. Classification of Cortical Demyelination

**Figure 1:**
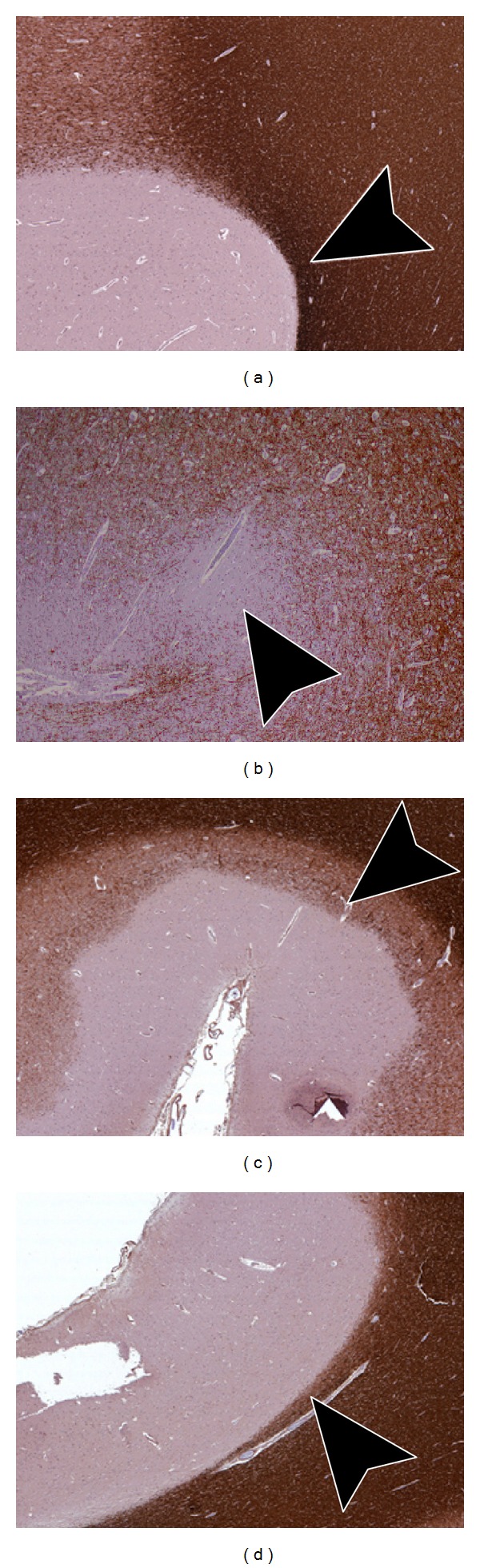
Pathologic classification of cortical lesions. (a) Type 1 “Leukocortical” lesions involve the deeper layers of gray matter and underlying white matter. Arrow head demonstrates the portion of the lesion extending into the white matter. (b) Type 2 “Intracortical” lesions are small lesions completely contained within the cortex, typically centered around small blood vessels. (c) Type 3 “Subpial” lesions extend from the pial surface into the superficial cortical layers, not reaching the white matter. (d) Type 4 “Subpial” lesions involve all layers of the gray matter but do not involve underlying white matter.

The initial classification system proposed for cortical demyelinating lesions subdivided lesions into seven types: a classification based on the relationship of lesions to the cerebral cortical venous supply [9]. Peterson et al. (2001) devised a less complex classification system based on the location of lesions within the layers of the cortex [10]. In this new system, there were three, rather than seven types. Type 1 “leukocortical” lesions involve the deep layers of gray matter and underlying white matter. Type 2 “intracortical” lesions are demyelinated lesions entirely contained within the cortex, not reaching the pial or subcortical white matter surface. Type 3 lesions extend from the pial surface into the superficial cortical layers. Bo et al. further refined this classification system with the addition of a fourth type: demyelination of all layers of the cortex but not the underlying white matter, typically involving multiple gyri [7]. Under this classification, both Type 3 and Type 4 lesions are “subpial.” Examples of the 4 lesion types are shown in Figure 1. Subpial Type 3 lesions are the most common, accounting for up to 60% of the total number of cortical lesions and 67% of the total lesion area. The leukocortical Type 1 and intracortical Type 2 lesions are the next most common. Subpial Type 4 lesions are the least common but cover the largest surface area [7, 8, 11].

### 2.2. Distribution/Extent of Gray Matter Demyelination

Cortical demyelinating lesions involve approximately 15–30% of the cortical gray matter [7, 12, 13]. Cortical demyelinating lesions occur in the early phases of relapsing remitting multiple sclerosis (RRMS) [14]; in rare cases occurring before focal WM lesions have developed [15]. Lesions accumulate over time, and in the later stages of secondary progressive and primary progressive multiple sclerosis (SPMS & PPMS) they are larger and are present in greater numbers [16, 17]. In fact, in some extreme cases of SPMS, cortical demyelinating lesions may involve up to 68% of the total cortical area [16]. 

Cortical demyelination is clearly widespread, and while there are relatively little differences in the involvement of each brain lobe, in general, the frontal and temporal lobes are affected slightly more than the parietal or occipital lobes [8, 12]. Within the temporal lobe, the hippocampus can be heavily involved [18, 19]. Deep gray matter structures such as the caudate, putamen, globus pallidus, and thalamus can be affected [17, 20]. Gray matter in other structures can also be involved, most notably the cerebellum and spinal cord [13, 21, 22]. 

### 2.3. Pathogenesis of Gray Matter Pathology: Inflammation, Neurodegeneration, or Both?

Cortical lesions obtained from autopsy specimens demonstrate a marked lack of inflammation: they are characterized by a general absence of T-cell and B-cell lymphocytes, complement deposition, vascular inflammation, and blood-brain barrier disruption [7, 23]. This paucity of inflammation stands in stark contrast to the highly inflammatory nature of WM lesions, which contain thirteen times more lymphocytes and six times more microglia and macrophages than cortical lesions [10]. While the inflammatory cell content within cortical lesions at autopsy is quite low, most of the inflammatory cells that are present are part of the intrinsic CNS immune response: activated microglia [10]. While there is little inflammation within cortical specimens from autopsy, biopsies from patients in early MS suggest a different inflammatory picture, with extensive cortical lymphocytic infiltration seen in the cortex in some patients [14, 15]. 

Although the inflammatory profiles of cortical demyelinating lesions show marked differences between autopsy and early biopsy, prominent areas of meningeal inflammation have been demonstrated throughout the course of the disease. This meningeal inflammation is present both in early RRMS [14], and in SPMS [16, 24–26], and in PPMS [27]. In many instances, meningeal inflammation is associated with ectopic B-cell tertiary structures which are reported to be topographically associated with cortical lesions [25, 26]. Meningeal inflammation is most commonly, but not always, associated with subpial lesions (Type III/IV), and it is theorized that myelinotoxic agents may diffuse from the meninges into the superficial gray matter, possibly contributing to demyelination [25]. 

Given these findings, how then can one explain the differences between the scant inflammation found in cortical lesions reported on autopsy studies, the marked inflammation in cortical lesions in early MS, and the meningeal inflammation occurring throughout the course of the disease? It is possible that specimens from autopsy and biopsies are presenting at least a somewhat skewed picture of the inflammatory profiles of MS lesions: lesions requiring biopsy in early MS are much more inflammatory and aggressive than more typical MS lesions and autopsy specimens come predominantly from older patients with end-stage disease. It may be that there are changing inflammatory profiles at different stages of lesion development [28]. Work by Merkler et al. (2006) has shown that early intense inflammation within cortical demyelinating lesions in an EAE rat model resolves relatively rapidly [29]. Merkler et al. (2006) and Albert et al. (2007) have shown that cortical remyelination is quite prominent in the earlier stages of MS [12, 29]. By the time lesions are analyzed on autopsy, the inflammation within these cortical lesions may have waned. It remains to be seen whether inflammatory profiles are isolated to specific points in time in the evolution of MS, or if they might occur across all disease stages.

While cortical demyelination is clearly occurring, there is also evidence of damage to the neurons themselves: multiple studies show evidence of neuronal atrophy, apoptosis, decreased neuronal densities, and reduced synaptic and glial densities [10, 11, 30]. The close approximation of activated microglia and astrocytes to neurons in cortical lesions, with activated microglia ensheathing neuronal bodies and neuritic elements such as axons and dendrites in cortical lesions, suggests a prominent role for the intrinsic immune response and inflammatory cortical demyelination [10]. It has been suggested however that primary neurodegenerative processes may also be occurring and that they may be partially independent [11, 17, 21, 31, 32]. Mitochondrial dysfunction [33–35] and defects in ion channels [36] have been proposed as possible independent mechanisms for primary neurodegeneration in MS. The picture however is not clear. Other studies have shown that mitochondrial injury appears to be driven by inflammation—specifically inflammation-associated oxidative bursts in activated microglia and macrophages [37, 38]. In keeping with this, a human pathology study has demonstrated a direct relationship between neuronal injury, axonal injury, and inflammation, arguing that in human MS there is not a separate neurodegenerative process [39]. 

## 3. Neuroimaging of Gray Matter Pathology

Advanced neuroimaging techniques make it possible to assess the development and progression of gray matter pathology *in vivo* something impossible using immunohistochemical approaches from autopsy and early biopsies. These *in vivo* MRI approaches have inherent advantages and limitations. Despite these limitations, they allow assessment of the accumulation of gray matter pathology over the course of the disease, the correlation of this damage with clinical disability and cognitive impairment, and assessment of the efficacy of disease-modifying therapies on measures of gray matter pathology.

### 3.1. Imaging of Gray Matter Demyelination

**Figure 2:**
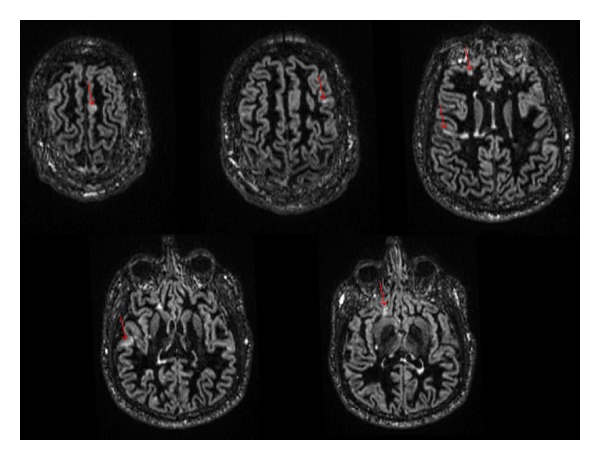
Cortical lesions in MS on Double Inversion Recovery. 41-year-old female patient with RRMS. Multiple axial DIR images demonstrate multiple small cortical and leukocortical lesions scattered in the supratentorial brain (red arrows).

Conventional MRI techniques for imaging MS are very insensitive for detection of focal cortical MS lesions: T2-weighted spin echo (T2-SE) and fluid attenuated inversion recovery (FLAIR) imaging identify around 2/3rds of pathologically identified WM lesions; yet respectively, they miss up to 97% and 95% of cortical lesions [6]. Double inversion recovery (DIR) improves on the performance of FLAIR and T2-SE imaging, detecting 538% more cortical lesions than T2-SE and 152% more cortical lesions when compared to FLAIR [40]. Examples of cortical lesions on DIR are shown in Figure 2.

Despite these gains, DIR is not without its drawbacks. As DIR suppresses signal from both WM and CSF, the resulting image has a low signal to noise ratio [41]. DIR is also prone to flow and pulsation artifacts and has relatively slow acquisition times [41–44]. These artifacts produce regional variations in gray matter signal intensity that can lead to errors in lesion detection [45]. While these deficiencies can be improved by certain multislab [43, 46] and single slab [47] 3D acquisitions, the SNR does not substantially improve [47]. The low SNR, image artifacts, and variations in DIR technique between institutions contribute to relatively large differences in agreement between observers when scoring cortical lesions, variation which persists even when using international consensus guidelines [48]. 

As such, DIR still misses the majority of cortical lesions [49, 50]. Seewann et al., performing postmortem confirmation of cortical demyelinating plaques, showed that 3D-DIR fails to identify 80% of pathologically confirmed cortical lesions, predominantly the purely cortical lesions (Type II–IV) [50]. In this study, DIR had a prospective sensitivity of 83% for leukocortical lesions (Type 1) and 7% for subpial lesions (Type III/IV). Despite the relative insensitivity of DIR for purely intracortical lesions, specificity was much higher at 90%. Interestingly DIR's sensitivity is greatest for lesions in the infratentorial brain [44]. Why are some lesions visible on MRI while others are not? Seewaan et al. have shown that cortical lesions that are visible on MRI are not histopathologically different from lesions invisible on MRI. They are however significantly larger, thus suggesting that lesion size, rather than underlying pathology, determines if a lesion is visible or not [49]. 

In order to further improve on the sensitivity and specificity of DIR for cortical lesions, other imaging techniques have been used in concert with DIR: for example, phase-sensitive inversion recovery (PSIR) [45] and T1-weighted 3D FSPGR (T1-3D-FSPGR) imaging [51]. PSIR preserves the sign of the magnetization, additively combining negative and positive magnetization, which effectively doubles the dynamic range of the image and provides superior gray white matter contrast [52]. Combined PSIR and DIR results in a 337% improvement in total number of cortical lesions detected as compared to FLAIR [45]: more than double the 150% improvement was seen when DIR is used alone [43]. Sethi et al. compared PSIR and DIR head to head in 60 MS patients and 30 controls and found that PSIR detected 3 times more intracortical lesions and 3 times more leukocortical and juxtacortical lesions than DIR [52]. The high SNR of T1-3D-FSPGR imaging is also useful in the detection of cortical lesions [51], primarily allowing greater anatomic specificity. Nelson et al. showed that the inclusion of T1-3D-FSPGR imaging improved lesion classification as compared to combined DIR PSIR, overturning the original classification in 30 of 119 lesions [53].

The ability to detect cortical lesions improves at higher field strengths. Compared to 1.5T, 3T DIR brain imaging identifies 192% more pure intracortical lesions and 30% more leukocortical lesions [54]. With ultrahigh field MRI (i.e., ≥7T), there are even more substantial improvements in SNR, spatial resolution, and image contrast, which results in increased detection of gray matter lesions [55–57]. The application of specialized 2D T2*-weighted gradient echo and 3D T1-MPRAGE sequences in ultra-high field MRI can produce high resolution anatomic images of cortical lesions [58–60]. Interestingly ultra-high field techniques appear to be very sensitive to subpial lesions (Type III/IV) [55, 59, 61], which are very difficult to detect at standard field strengths.

### 3.2. Quantification of Diffuse Injury in Normal Appearing Gray Matter (NAGM)

While focal cortical demyelination occurs in MS and is detectable with DIR and other advanced sequences, diffuse demyelination is also occurring in NAGM. Both conventional and more advanced techniques fail to identify abnormalities in the NAGM, even though this tissue may be histopathologically abnormal. Various quantitative MRI techniques have been utilized to assess for diffuse demyelination in NAGM. Of them, magnetization transfer imaging (MTI) and diffusion tensor imaging (DTI) have been the most studied. 

MTI measures the capacity of protons bound to macromolecules in brain parenchyma to exchange magnetization with free water [101]. The efficiency of this exchange is reflected in the magnetization transfer ratio (MTR). Reductions in the MTR provide a measure of the extent of damage to the microstructure of brain tissue and has been correlated with the degree of demyelination and axonal loss in MS patients [102]. Reduced MTR in NAGM is present in all MS clinical phenotypes [103–106] but is greatest in SPMS and PPMS [107]. Similar to neocortical gray matter, decreased MTR has been identified in deep gray matter structures, particularly the thalami [74, 108]. 

DTI enables the measurement of the random diffusional motion of protons in water, providing metrics such as mean diffusivity and fractional anisotropy [109] which correlate with demyelination and axonal loss. While typically DTI has been used to assess damage to normal appearing white matter, DTI can detect damage to myelin in the NAGM. In all stages of MS, longitudinal studies have demonstrated increased mean diffusivity values in the NAGM over time [70, 110, 111]. As is true for MTR, thalamic damage can be assessed, with studies showing higher mean diffusivity in the normal appearing thalamus of patients with MS as compared to controls [77].

### 3.3. Imaging of Gray Matter Atrophy

**Figure 3:**
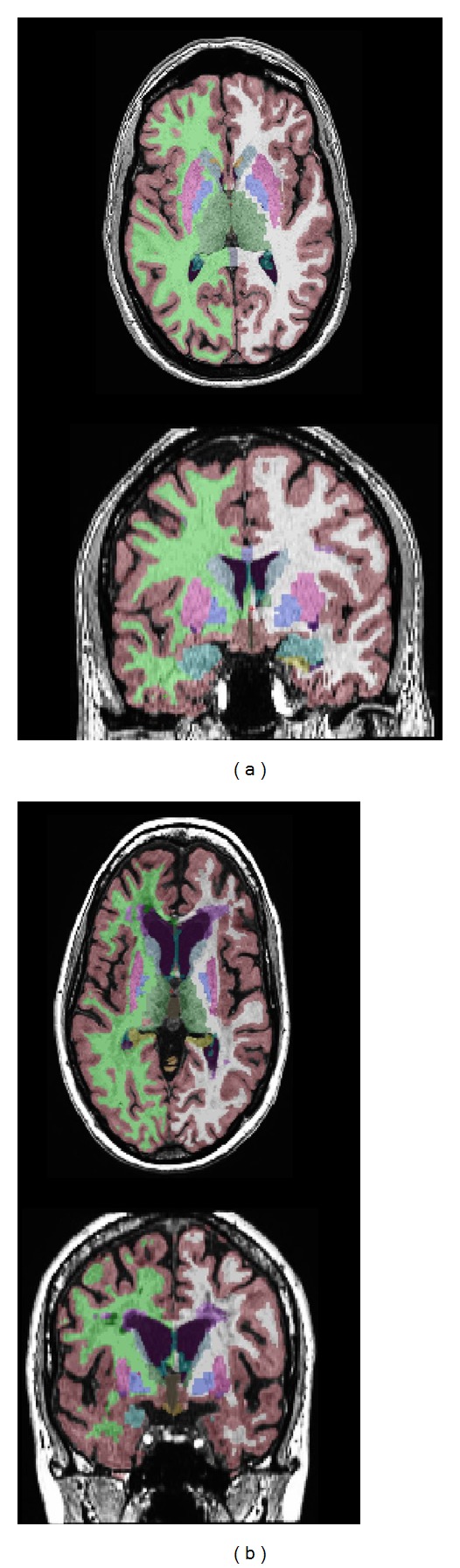
Atrophy in multiple sclerosis as measured using Freesurfer (http://surfer.nmr.mgh.harvard.edu/). (a) Cortical and subcortical segmentation in a healthy control patient. (b) Cortical and subcortical segmentation in an age-matched patient with longstanding RRMS. Note the prominent subcortical, white matter, and neocortical atrophy in MS compared to healthy control.

Brain atrophy in multiple sclerosis patients begins at disease onset in CIS, progressing throughout the course of the disease with the greatest accumulation of atrophy in long standing SPMS [112–115]. Atrophy can be quite prominent, as is demonstrated in Figure 3. While both gray matter and white matter tissues are being lost, it is the gray matter atrophy which appears to predominate: Fisher et al. demonstrated progressively increased rates of gray matter atrophy in the various stages of MS but no change in rate of white matter atrophy over time [83]. Similar findings were demonstrated by Fisniku et al. [84]. Indeed, gray matter and white matter atrophy may be partially independent [114, 116] suggesting a role for both inflammatory gray matter demyelination, retrograde neuronal loss from white matter injury, and possibly neurodegenerative processes in the evolution of gray matter atrophy over time. 

Measurement of gray matter atrophy has several advantages over detection of cortical lesions. Gray matter lesions are difficult to visualize even with advanced sequences, and there is significant variation between readers. Gray matter atrophy measurements on the other hand, are more reliable and the results are reproducible among research institutions [117, 118]. 

While manual segmentation by trained expert readers is the gold standard for quantification of atrophy, the difficulty and time-consuming nature of manual segmentation precludes its use on large numbers of patients. As such, computer-assisted techniques are used. In general, these techniques can be divided into those that *segment* the brain into its constituent parts and those which use *registration* and then image *subtraction* to directly quantify brain volume changes between two time points [119]. Two of the most commonly used registration-based techniques are the brain boundary shift integral (BBSI) [120] and the structural image evaluation using normalization of atrophy (SIENA) [121]. These registration techniques measure total brain volume changes; they do not specifically measure gray matter volume, rather a combination of grey and white matter atrophy. Segmentation techniques on the other hand allow quantification of total or regional gray matter, cortical gray matter, and deep grey matter volumes. Three of the most commonly reported techniques are (1) voxel-based morphometry (VBM) using the statistical parametric mapping (SPM) software suite [122], (2) SIENAx as part of the FSL software library [118], and (3) Freesurfer [123]. There are a large number of other software packages available, commercial, open source, and custom software used by specific institutions.

While these software packages can perform their analysis on a variety acquired sequences, isotropic, high resolution T1 weighted 3D volumetric acquisitions are best able to detect the small changes in atrophy which occur over time [124]. This atrophy is usually measured as changes in volumes of gray matter structures. Volumes can be expressed as absolute volumes, but due to baseline variations in head size, normalization is necessary to compare patients. Normalization can be attained via a number of different processes: the most common being normalization to intracranial volume (as is done for the BPF, GMF, and WMF), skull size, or by transformation to standard space. Cortical thickness can also be measured both with VBM [122] and Freesurfer [123]; VBM assesses cortical thickness changes between groups of patients while Freesurfer measures thickness in each individual. With cortical thickness measurements, as with volume measurements, either global or regional changes can be assessed. 

Measures of gray matter volume and cortical thickness depend heavily on the appropriate classification of gray and white matter, but in general the available software is not specifically designed to account for the similarity of signal intensities of white matter lesions on T1-weighted images to that of normal gray matter. As such, this leads to errors in gray matter tissue classification [122, 125–127]. This tissue misclassification occurs with all types of white matter lesions but can be especially problematic in patients with substantial subcortical disease. Lesion in-painting, where white matter lesions are prospectively masked before tissue segmentation, significantly improves gray matter segmentation [128, 129] and should be implemented in future studies to ensure reliability of volume measurements.

## 4. Gray Matter Pathology and Diagnosis of MS

Neuroimaging studies have clearly demonstrated that cortical lesions occur in all phenotypes of multiple sclerosis [42], occurring not just in the late stages of disease, but early on as well. In fact cortical lesions have been visualized before white matter lesions have developed [15, 130], as well as in both radiologically isolated syndrome [131] and clinically isolated syndrome [42]. Similar to cortical demyelination, gray matter atrophy is detectable very early in the disease and accelerates over time [87, 90, 114]. 

If both cortical atrophy and demyelination are occurring before a diagnosis of clinically definite MS can be made, how then can measures of cortical lesions and cortical atrophy be best applied to make an early and accurate diagnosis of MS? Filippi et al. have shown that the addition of DIR to detect cortical lesions makes small but significant improvement to the accuracy of conventional MRI diagnostic criteria (81% accuracy versus 75–78% accuracy with traditional criteria) [132]. The presence of gray matter lesions may be predictive of conversion to MS: Filippi et al., showed that a patient with clinically isolated syndrome with ≥1 intracortical lesion had an odds ratio of 15.3 for conversion to clinically definite MS [132]. As with cortical lesions, gray matter atrophy measures appear to be predictive: Calabrese et al., demonstrated that compared to CIS patients meeting criteria for dissemination in space, patients with CIS and atrophy of either the superior frontal gyrus, thalamus, or cerebellum had double the risk of conversion to clinically definite MS [90]. It should be noted, however, that the predictive power of gray matter atrophy may be less than gray matter lesions [90]. While the presence of gray matter lesions may be more predictive of conversion to MS than atrophy, one must keep in mind the reliability of measures of both these types of pathology. As mentioned previously, Geurts et al. showed that the coefficient of variation between gray matter lesion counts with DIR was quite large (42%), even when using consensus guidelines [48]. The reliability of measures of gray matter volumes are substantially better, with coefficients of variation in gray matter volumes of 1% or less [133]. While it is clear that the addition of measures of gray matter pathology to diagnostic criteria for MS is likely to improve accuracy, it is as of yet established what specific MRI measures of the gray and white matter disease are optimal to predict conversion to MS in patients with RIS or CIS. 

## 5. Effect of Gray Matter Lesions on Disability and Cognition

Gray matter lesion burden has been correlated with clinical disability in all phenotypes of MS (Table 1). Calabrese et al. have demonstrated that increased numbers of intracortical lesions accumulate in later stages of the disease, with 36% of CIS, 64% of RRMS, and 73% of SPMS patients having detectable cortical lesions [42]. In this study, the number of intracortical lesions was moderately correlated with higher Expanded Disability Status Scale Scores (EDSS) (*r* = 0.48, *P* = 0.001) [42]. Mike et al. have shown similar correlation between lesion number and EDSS (*r* = 0.472, *P* ≤ 0.05) [66] and others have shown such a correlation between lesion number and physical disability in PPMS [134].

Accumulations of greater numbers of lesions are not the whole picture, as both lesion volume and the rate of accumulation of cortical lesions appear to play a role [66]. In a 3-year longitudinal study, Calabrese et al. found that cortical lesion volume was the best independent predictor of worsening EDSS scores (*r* = 0.55 in RRMS and *r* = 0.43 in SPMS (*P* ≤ 0.001)); cortical lesion volume and cortical lesion numbers increased over time faster in patients with disability progression than those who were stable [65]. In a 5-year longitudinal study of MS patients cortical lesion volume was an independent contributor to EDSS, and patients with clinical progression accumulated cortical lesions the fastest [69]. Conversely in studies of benign MS (EDSS < 3, 15 years from clinical onset), Calabrese et al. showed that patients with benign MS had fewer cortical lesions, smaller lesion volumes, and slower accumulation of cortical lesions than patients with RRMS with similar disability scores [62, 135]. In these studies, the best predictors of benign disease were smaller volumes of cortical lesions and slower rate of increase over time.

Using a variety of cognitive tests such as the Paced Auditory Serial Addition Test (PASAT), the Symbol Digit Modality Test (SDMT), and Rao's Brief Repeatable Battery (RBRB), cortical lesions have also been shown to be associated with cognitive impairment (Table 1). Attention deficits and slowing of information processing speed are the most commonly observed impairments. Various aspects of executive function are frequently affected [136]. As is true with physical disability, increases in both lesion number [63, 69] and lesion volume [64, 66, 67, 69, 137] are correlated with cognitive impairment. 

## 6. Effect of Diffuse Injury to NAGM on Disability and Cognition

Studies of MTR reduction in NAGM show clear correlation with progression of clinical disability and cognitive impairment (Table 2). Fisniku et al., assessing 69 patients 20 years after they originally presented with CIS, demonstrated that reduced peak height MTR of gray matter was both the best independent predictor of disability as measured by EDSS and the only independent predictor of cognitive impairment as measured by the MSFC [76]. Agosta et al. have shown that reduction of MTR peak height in gray matter is an independent predictor of accumulation of disability over 8 years [72]. In benign MS, Amato et al. found correlations between reduced cortical MTR and measures of cognition [75]. Regional rather than diffuse changes in MTR in NAGM may also be important. Khaleeli et al., studying 46 patients with PPMS, demonstrated that EDSS correlated with lower MTR in the right primary motor cortex, and poorer performance on PASAT correlated with MTR in the right inferior parietal and inferior occipital cortices [74]. There were no correlations in other regions. Similar correlations between PASAT performance and regional MTR reductions in the parietal lobe (left BA40) have also been demonstrated in RRMS [71].

Similar to MTR, alterations in mean diffusivity in the NAGM of MS patients are correlated with clinical disability and cognitive impairment. Rovaris et al. have shown that average gray matter mean diffusivity is correlated with the degree of cognitive impairment as measured by the SDMT [70]. In PPMS, average gray matter mean diffusivity was an independent predictor of subsequent clinical deterioration over 5 years as measured by changes in EDSS scores [73]. Elevated gray matter mean diffusivity changes in the thalami correlate with both PASAT performance (*r* = −0.42, *P* = 0.034) and the EDSS (*r* = 0.47, *P* = 0.021) [77].

## 7. Effect of Gray Matter Atrophy on Disability and Cognition

Gray matter atrophy is significantly correlated with both physical and cognitive disability in MS patients [64, 80–95, 138, 139]. These correlations are found at all stages of disease and have the strongest correlation in SPMS. While correlations are strongest in late stages of the disease, cortical atrophy appears relevant even before clinical symptoms are evident: Amato et al. have shown that in asymptomatic patients with radiologically isolated syndrome, 27.6% of patients have signs of cognitive impairment similar to those of RRMS and low normalized cortical volumes were associated with a higher number of failed cognitive tests [93].

While gray matter atrophy in MS is a global and widespread phenomenon, it appears that the location of atrophy (i.e., what specific brain region is affected) may be as important as global changes in gray matter volumes. Calabrese et al. have demonstrated that cortical thinning in the precentral gyrus is strongly and significantly correlated with motor Functional System Scores in RRMS (*r* = −0.626, *P* < 0.001) [114]. Cortical gray matter thinning in the parietal and precentral gyri is significantly correlated with disability progression as measured by MSFC and EDSS, respectively [79]. Using various cognitive tests, localized cortical atrophy in the prefrontal, parietal, temporal and insular regions has been associated with deficits in attention, information processing speed and verbal memory [81, 92]. Regional atrophy plays a role not just in cerebral cortex, but also in the cerebellum and deep gray matter structures. Anderson et al. have demonstrated that cerebellar gray matter volumes are significantly smaller in patients with cerebellar dysfunction than in those with normal cerebellar function [140]. The deep gray matter volumes (basal ganglia and especially the thalami) are correlated with disability and cognitive impairment: particularly significant are deficits in information processing speed [82, 94], but fatigue [89] and EDSS scores [86, 90] are also correlated. When gray matter atrophy and other MRI parameters such as T2 lesion volume, T1 lesion volumes, and white matter atrophy, are measured, gray matter atrophy is the strongest correlate of cognitive and clinical disability. Examples of the studies showing correlation between gray matter atrophy and disability are shown in Table 3. 

## 8. Effect of Disease-Modifying Therapies on Gray Matter Pathology

Although the interrater variability in cortical lesion counts on DIR imaging limits its utility for large multicenter clinical trials, single center data where imaging techniques and raters are standardized appear to demonstrate that disease-modifying therapies (DMT) slow the development of cortical lesions in MS (Table 4). Calabrese et al., in a 2-year randomized study demonstrated that both interferon beta-1a (IFN-beta-1-a) and glatiramer acetate (GA) therapy resulted in significantly decreased new cortical lesion development (at 1 year 74% of untreated patients had new cortical lesions versus 45% of treated patients) [99]. The effect was greatest for high dose, high frequency subcutaneous IFN beta-1a. Natalizumab also appears to slow the rate of cortical lesion development: Rinaldi et al. demonstrated that after 1 year of therapy only 14% of treated patients had developed at least one new cortical lesion [141]. At two years, 20% of natalizumab patients had a new cortical lesion compared with 74% of untreated patients [100]. The effect of natalizumab on new cortical lesion development was stronger than that of other first line immunomodulatory agents (IMAs) (IFN-beta-1a or GA) with on average 0.2 new cortical lesions in natalizumab treated patients and 1.3 new cortical lesions in the group treated with other IMAs [100].

The improved reliability of gray matter atrophy measurements and standardization of procedures across institutions has allowed evaluation of the effect of DMTs on measures of gray matter atrophy. Tiberio et al. showed no effect on gray matter atrophy at 1 year for patients treated with IFN-b [142]; however, more recent studies have shown positive effects of therapy. Zivadinov et al., in a nonrandomized open label study, demonstrated that intramuscular IFN-beta-1-a significantly slowed the progression of whole brain and gray matter atrophy compared with untreated patients (−1.3% BPF in treated patients versus −2.5% BPF in untreated patients) [96]. Nakamura et al. [97] have shown similar results. Calabrese et al. [99] did not show differences in the rate of decrease in gray matter fraction between therapeutic groups; however, Bendfeldt et al. [98] demonstrated slowing of atrophy, with progression of regional gray matter atrophy differing between patients treated with different IMAs [98]. Most recently Rinaldi et al. has shown slowing of cortical atrophy/thinning with both IFN-b and GA (3.7% global cortical thickness loss in treated patients versus 4.6% in untreated patients over 2 years) [100]. As with cortical lesions, natalizumab has a stronger effect on the slowing of cortical atrophy than other IMAs: natalizumab had the lowest rate of atrophy at 1.7% cortical thickness loss over 2 years. This thinning was not statistically different when compared to healthy controls [100]. 

While these studies indicate that DMTs slow rate of accumulation of gray matter lesions and gray matter atrophy in MS, the nonrandomized nature of the bulk of these studies somewhat limits their impact. Currently, no disease-modifying therapy available for treating MS patients have completely stopped the evolution from RRMS to the progressive phase of the disease. However, highly active therapies, such as natalizumab, may have a greater impact on the accumulation of gray matter damage than first line agents [100, 142]. Randomized studies investigating the effects of longer term treatment with highly active therapy (>2 years) will provide further insight into their ability to alter the course of MS.

## 9. Conclusions

As is clear from pathologic studies and neuroimaging, gray matter pathology is of critical importance in multiple sclerosis. Despite the advances that have been made, it remains unclear as to what are the underlying causes of gray matter pathology, and what the exact relationship is between gray matter demyelination and measures of diffuse gray matter damage and atrophy. Some have suggested that primary neurodegenerative process occurring in MS may be at least partially independent inflammation, while other studies have shown a direct relationship between neuronal and axonal injury and inflammation. The specifics of these interactions need to be fully investigated as this has significant implications for therapeutic design. If independent neurodegenerative processes are occurring in MS, neuroprotective therapies will be vitally important; however, if inflammation drives subsequent neurodegeneration, anti-inflammatory therapies would be the best choice for patients.

The improved *ex vivo* detection of gray matter pathology in MS has spurred research which has substantially improved the detection of gray matter damage *in vivo* with MRI. It is now possible to directly visualize focal cortical lesions using a variety of sequences such as DIR, PSIR, and 3D T1-FSPGR. Ultra-high field MRI techniques provide even greater anatomic delineation of lesions and allows for improved anatomic classification. Subtle changes in gray matter below the threshold of conventional MRI can be assessed by quantitative MR techniques such as MTI and DTI. Additionally gray matter atrophy can now be reliably measured using automated and semiautomated computerized techniques. Despite these advances, imaging of gray matter pathology remains quite difficult, as even advanced sequences such as DIR fail to identify the vast majority of gray matter lesions, and there is considerable variability in lesion identification rates between observers. Gray matter atrophy measures appear to be more reproducible and reliable but the similarity between MS lesions and gray matter on T1 weighted images leads to errors during segmentation. Greater standardization of methods for the measurement of gray matter atrophy and cortical lesion delineation are needed to improve our understanding of the relationship between gray matter pathology and the disease process. 

Despite these limitations, researchers using these techniques have amassed a large body of data which clearly shows that there is an association between various measures of gray matter pathology, clinical disability, and cognitive impairment. Both neocortical gray matter and deep gray matter structures are affected, with both global (diffuse) and focal gray matter injury playing a role. Impairment likely relates to both disruptions of large scale cortical networks and focal injuries to areas critical for specific functions. It remains to be determined if specific imaging modalities are better able to explain clinical symptoms as compared to other modalities and what measures and modalities best predict the conversion to MS. 

previously As detailed there are many unanswered questions regarding the role of gray matter pathology in MS and the optimal applications of MR imaging modalities in the diagnosis, monitoring, and treatment of MS patients. Over the past decade and a half, there have been incredible advances in our understanding of the role of gray matter pathology in MS. These advances have begun to answer these questions but further work is needed and is ongoing. 

## Figures and Tables

**Table 1:** Neuroimaging studies evaluating the relationship between cortical lesions, clinical disability and cognitive impairment.

Study	Method	Number of patients	Relevant findings
Calabrese et al. 2007 [42]	Cross sectional 1.5T; DIR	116 CIS, 163 RRMS, 101 SPMS 40 HC	Cortical lesions occur in greater numbers in SPMS than in CIS or RRMS. Cortical lesion numbers are correlated with EDSS.
Calabrese et al. 2009 [62]	1 yr longitudinal 1.5T; DIR	48 Benign MS 96 RRMS	Benign MS patients have lower numbers of cortical lesions and lower cortical lesion volumes as compared to early RRMS, and do not accumulate a statistically significant number of new lesions over 1 year.
Roosendaal et al. 2009 [63]	3 yr longitudinal 1.5T; DIR	9 RRMS, 4 SPMS 7 HC	Cortical lesions increase significantly in MS patients over time and are associated with worse performance on neuropsychological measures. Cortical lesions are most frequent in SPMS.
Calabrese et al. 2009 [64]	Cross sectional 1.5T; DIR	70 RRMS	Cortical lesion volume and number correlate with most, but not all, of the cognitive tests of Rao's brief repeatable battery. Cortical lesion volume is an independent predictor of the cognitive impairment index.
Calabrese et al. 2010 [65]	3 yr longitudinal 1.5T; DIR	76 RRMS, 31 SPMS	Baseline cortical lesion volume best predicted disability progression (as measured by EDSS) over the follow-up period.
Mike et al. 2011 [66]	Cross sectional 3T; 3D FLAIR & 3D IRSPGR	20 RRMS, 6 SPMS	Cortical lesion number and volume independently predicted EDSS. Cortical lesion number and volume independently predicted performance measured by the Symbol Digit Modality Test. Only cortical lesion number predicted performance measured by the California Verbal Learn Test.
Nelson et al. 2011 [67]	Cross sectional 3T; DIR/PSIR	39 MS (Subtypes not specified)	Leukocortical lesion numbers are independently correlated with cognitive impairment, while purely intracortical lesions are not independent contributors. The size of cortical lesions, not the tissue specific location, may better explain the correlation with cognitive impairment.
Calabrese et al. 2012 [68]	3 yr longitudinal 1.5T; DIR	32 RRMS with epilepsy, 60 RRMS without epilepsy	Cortical lesion number and volumes are larger in patients with epilepsy than those without. RRMS patients with epilepsy accumulate lesions at a faster rate than nonepileptic patients over 3 years.
Calabrese et al. 2012 [69]	5 yr longitudinal 1.5T; DIR	157 RRMS, 35 Pediatric MS, 45 Benign MS, 44 PPMS, 31 SPMS	Higher cortical lesion loads correlate with higher EDSS. Patients with clinical progression have the highest rate of cortical lesion accumulation. Cortical lesion volumes are independent predictors of disability progression and cognitive impairment.

**Table 2:** Neuroimaging studies assessing the relationship between markers of diffuse damage in NAGM and clinical disability and cognitive impairment.

Study	Method	Number of patients	Relevant findings
Rovaris et al. 2002 [70]	Cross sectional 1.5T; DTI	34 RRMS	Average gray matter mean diffusivity correlates with cognitive impairment measured by the Symbol Digit Modality Test (SDMT).
Ranjeva et al. 2005 [71]	Cross sectional 1.5T; MTR	18 CIS 18 HC	Decreased MTR in the parietal lobe (BA40) correlates with poorer performance on Paced Auditory Serial Addition Test (PASAT).
Agosta et al. 2006 [72]	8-year longitudinal 1.5T; MTR	73 MS (34 RRMS, 19 SPMS) 20 CIS 16 HC	Lower gray matter MTR-peak height at baseline is an independent predictor of accumulation of disability over 8 years as measured by the Expanded Disability Status Scale (EDSS).
Rovaris et al. 2006 [73]	5-year longitudinal 1.5T; DTI	55 PPMS	Average gray matter mean diffusivity was an independent predictor of clinical deterioration as measured by EDSS.
Khaleeli et al. 2007 [74]	Cross sectional 1.5T; MTR	46 PPMS 23 HC	Lower MTR in the right primary motor cortex correlated with disability as measured by EDSS. Lower MTR in the right parietal and occipital cortices correlated with poorer performance on PASAT.
Amato et al. 2008 [75]	Cross sectional 1.5T; MTR	47 Benign MS 24 HC	Reduced cortical MTR correlated with a variety of measures of cognitive impairment.
Fisniku et al. 2009 [76]	Cross sectional 1.5T; MTR	41 MS (31 RRMS, 10 SPMS) 28 CIS 19 HC	Reduced gray matter peak height MTR is the best independent predictor of disability as measured by EDSS, and only independent predictor of cognitive impairment as measured by the Multiple Sclerosis Functional Composite (MSFC).
Tovar-Moll et al. 2009 [77]	Cross sectional 3T; DTI	24 MS (13 RRMS, 11 SPMS) 24 HC	Elevated mean diffusivity within the thalamus correlated with performance on PASAT and EDSS.
Crespy et al. 2011 [78]	Cross sectional 1.5T; MTR	88 CIS 44 HC	Gray matter MTR decrease is significantly associated with worse EDDS scores. (*R* ^2^ = 0.135, *P* = 0.002).

**Table 3:** Neuroimaging studies evaluating the relationship between gray matter atrophy, clinical disability, and cognitive impairment.

Study	Method	Number of Patients	Relevant Findings
Chen et al. 2004 [79]	1 yr longitudinal 1.5T; cortical thickness	24 RRMS, 6 SPMS	Cortical thickness decreased 3.13% ± 2.88%/year in patients with progressive disability. In stable patients 0.06 ± 2.31%/year change in cortical thickness.
Tedeschi et al. 2005 [80]	Cross sectional 1.0T; GMF	427 RRMS, 140 SPMS 104 HC	GMF is the most significant MRI variable in determining final disability as measured by EDSS.
Morgen et al. 2006 [81]	Cross sectional 1.5T; NGMV	19 RRMS 19 HC	Patients with low cognitive performance showed more extensive cortical volume loss than HC in the frontal, temporal, and parietal lobes.
Houtchens et al. 2007 [82]	Cross sectional 1.5T; NTV	26 RRMS, 5 SPMS	Cognitive performance in all domains was correlated with thalamic volume in MS group (*r* = 0.506–0.724, *P* < 0.005) and EDSS (*r* = −0.316, *P* = 0.005).
Fisher et al. 2008 [83]	4 yr longitudinal 1.5T; GMF	7 CIS, 36 RRMS, 27 SPMS 17 HC	GMF correlated with both the MSFC and EDSS. Increasing contribution of GM atrophy to whole brain atrophy as MS advances.
Fisniku et al. 2008 [84]	Cross sectional 1.5T; GMF	29 CIS, 33 RRMS, 11 SPMS 25 HC	GMF, not white matter volume, correlated with clinical disability as measured by EDSS and MSFC.
Horakova et al. 2009 [85]	5 yr longitudinal 1.5T; NGMV	181 Early RRMS 27 HC	NGMV and age were the best predictors of progression of EDSS.
Rocca et al. 2010 [86]	8 yr longitudinal 1.5T; NTV	20 CIS, 34 RRMS, 19 SPMS 13 HC	Baseline thalamic atrophy significantly correlates with deterioration in EDSS score.
Audoin et al. 2010 [87]	Cross sectional 1.5T; regional GMV	62 CIS 37 HC	Right cerebellar atrophy correlated with EDSS scores but no correlation between regional atrophy and cognitive status.
Calabrese et al. 2010 [88]	Cross sectional 1.5T; Cortical thickness	100 RRMS 42 HC	A widespread pattern of cortical thinning is the best predictor of cognitive impairment as measured by the Rao's Brief Repeatable Battery
Calabrese et al. 2010 [89]	Cross sectional 1.5T; Cortical thickness, DGMV	152 RRMS 42 HC	Significant atrophy of striatum, thalamus, superior frontal gyrus, and inferior parietal gyrus in fatigued patients compared to nonfatigued patients
Calabrese et al. 2011 [90]	4 yr longitudinal 1.5T; Cortical thickness	105 CIS 42 HC	CIS with atrophy of the superior frontal gyrus, thalamus, and/or cerebellum doubled the risk of conversion to MS.
Roosendaal et al. 2011 [91]	Cross sectional 1.5T; NGMV	95 CIS, 657 RRMS, 125 SPMS, 50 PPMS	NGMV was the strongest predictor of disability and cognitive impairment as measured by EDSS and PASAT.
Nocentini et al. 2012 [92]	Cross sectional 1.5T; GMF, regional GMV	13 RRMS, 5 SPMS	Significant associations found between scores on the SDMT and LDCR-CVLT with regional GM atrophy in prefrontal, parietal, temporal, and insular cortex
Amato et al. 2012 [93]	Cross sectional 1.5T; NCV	29 RIS, 26 RRMS 21 HC	In RIS, lower NCV correlated with worse cognitive performance.
Batista et al. 2012 [94]	Cross sectional 3.0T; NCV, NDGMV	59 RRMS, 27 SPMS	Both NCV and deep gray matter volumes are significantly correlated with cognitive impairment. Thalamic atrophy plays significant role in IPS slowing.
Zivadinov et al. 2013 [95]	2 yr longitudinal 1.5T; NCV	136 RRMS	Significant cortical atrophy occurs in early RRMS over 2 years and is associated disability progression.

Label: IPS: information processing speed. GMF: gray matter fraction. GMV: gray matter volume. MSFC: multiple sclerosis functional composite. NCV: normalized cortical volume. NDGMV: normalized deep gray matter volume. NGMV: normalized gray matter volume. NTV: normalized thalamic volume. RIS: radiologically isolated syndrome. SDMT: symbol digit modality test. LDCR-CVLT: long delayed cued recall-California Verbal Learning Test.

**Table 4:** Studies demonstrating the effects of disease modifying therapies on measures of gray matter pathology.

Study	Method	Number of patients	Duration	Relevant findings
Zivadinov et al. 2007 [96]	Nonrandomized. Intramuscular IFN beta-1a versus untreated patients	54 RRMS	3 years	IFN beta-1-a slows the rate of gray matter atrophy compared to untreated patients.
Nakamura et al. 2010 [97]	Randomized. Intramuscular IFN beta-1a versus placebo	131 RRMS	2 years	IFN beta-1a slows the rate of gray matter atrophy compared to placebo.
Bendfeldt et al. 2010 [98]	Nonrandomized. Subcutaneous IFN beta-1a versus intramuscular IFN beta-1a versus untreated patients	86 RRMS	2 years	IFN beta-1a reduced gray matter atrophy rates, while glatiramer acetate did not. Progression of regional gray matter volume loss differs between patients treated with different immunomodulatory agents.
Calabrese et al. 2012 [99]	Randomized. Subcutaneous IFN beta-1a versus intramuscular IFN beta-1a versus glatiramer acetate	141 RRMS	2 years	Effect of subcutaneous IFN beta-1a in preventing new cortical lesions was higher compared to both intramuscular IFN beta-1a and glatiramer acetate. Gray matter fraction decrease did not differ significantly among treatment groups.
Rinaldi et al. 2012 [100]	Nonrandomized. Natalizumab versus other IMAs (subcutaneous IFN beta-1a, intramuscular IFN beta-1a, or glatiramer acetate)	120 RRMS	2 years	Natalizumab treatment results in greater decreases in the rate of accumulation of cortical lesions and the progression of cortical atrophy as compared to other immunomodulatory agents.
